# LH/hCG-Receptor Expression May Have a Negative Prognostic Value in Low-Risk Endometrial Cancer

**DOI:** 10.3389/fonc.2016.00190

**Published:** 2016-08-25

**Authors:** Ivo Noci, Flavia Sorbi, Luca Mannini, Elisabetta Projetto, Serena Pillozzi, Viola Ghizzoni, Tiziano Lottini, Daniela Moncini, Gianna Baroni, Francesco Mungai, Annarosa Arcangeli, Massimiliano Fambrini

**Affiliations:** ^1^Department of Biomedical, Clinical and Experimental Sciences, University of Florence, Florence, Italy; ^2^Department of Experimental and Clinical Medicine, University of Florence, Florence, Italy; ^3^Department of Diagnostic Imaging, Careggi University Hospital, Florence, Italy

**Keywords:** endometrial cancer, biomarker, LH/hCG-receptor, recurrence, immunohistochemical analysis, molecular analysis

## Abstract

**Introduction:**

A 51 year-old woman was diagnosed with endometrial cancer (EC) and underwent surgical staging. Pathological evaluation showed a 2 cm × 1 cm G2 endometrioid EC with a 30% myometrial deep invasion (FIGO Stage 1A). The patient was classified as low risk of recurrence, and no adjuvant treatment was offered. Six months after surgery, the patient developed an early vescico-vaginal recurrence, and chemotherapy treatment was started. Few months later, a subsequent involvement of vaginal wall, ileum, and omentum was detected, and the patient underwent second surgery.

**Background:**

LH/hCG-receptor (LH/hCG-R) expression has been previously reported to be associated with an invasive phenotype in EC cells. Moreover, in a preclinical mouse model of EC behaves as a prometastatic molecular device.

**Discussion:**

We analyzed the expression level of LH/hCG-R in cancer specimens collected during surgeries. Molecular and immunohistochemical analyses showed a strong expression of both mRNA and protein for LH/hCG-R in all specimens.

**Conclusion:**

LH/hCG-R expression may be assessed together with other clinicopathological parameters in order to better predict the risk of recurrence in low-risk EC patients. Further clinical trials are warranted in order to validate LH/hCG-R as biomarker in EC.

## Introduction

**Figure 1:**
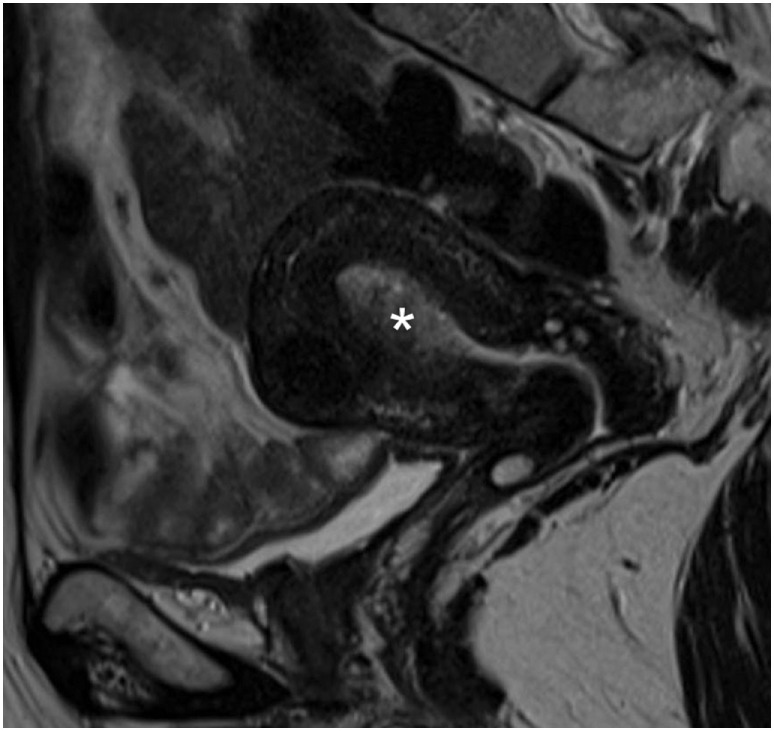
**Pelvic magnetic resonance image**. Sagittal T2-weighted magnetic resonance image showing endometrial cancer as heterogeneous intermediate-signal intensity lesion (white asterisk) disrupting the normal high-signal intensity endometrium and infiltrating less than half of the ventral myometrial thickness (revised FIGO IA Stage).

**Figure 2:**
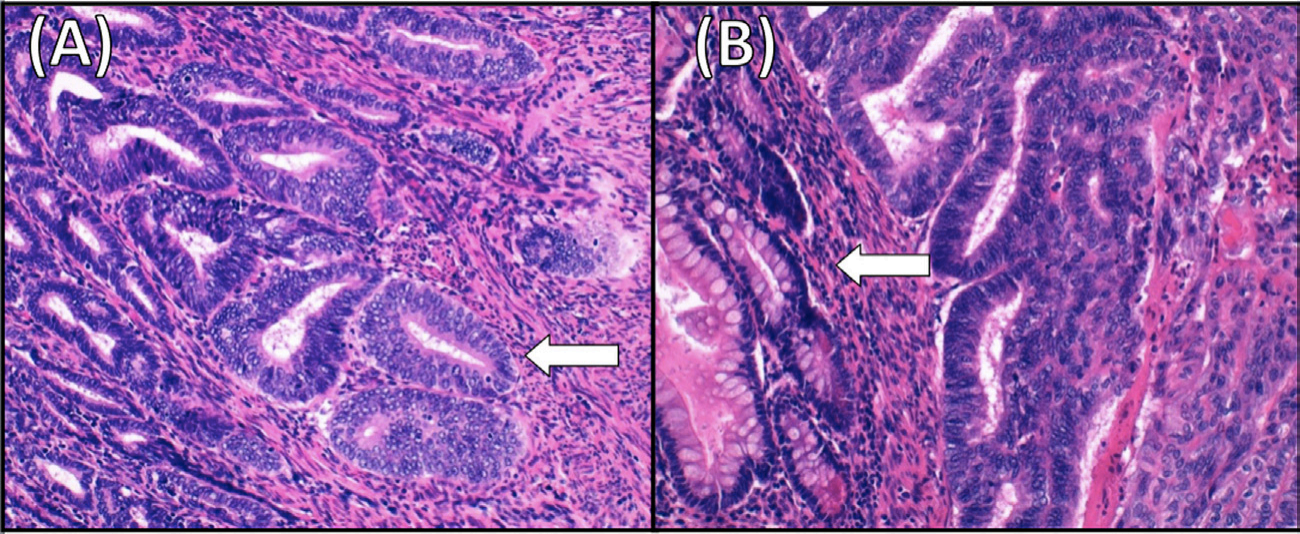
**Histology of the primary endometrial cancer and the ileum recurrence**. **(A)** Moderately differentiated endometrioid adenocarcinoma (G2). Superficial myometrial invasion of the tumor (arrow) (hematoxylin–eosin staining, ×20). **(B)** Endometrioid adenocarcinoma metastating to the ileum. The tumor invades bowel mucosa (arrow) and stroma connective adipose subserous (hematoxylin–eosin staining, ×20).

A 51-year-old postmenopausal woman presented with abnormal vaginal bleeding in December 2014. Ultrasound examination revealed a polypoid lesion in the uterine fundus. Office hysteroscopy with biopsy was performed and revealed a G2 endometrioid adenocarcinoma of the endometrium. A pelvic magnetic resonance imaging (MRI) demonstrated in T2-weighted image a 2 cm × 2.5 cm area of heterogeneous intermediate-signal intensity lesion disrupting the normal high-signal intensity endometrium and invading less than half of the myometrium (Figure 1). Then, in January 2015, she underwent hysterectomy and bilateral oophorectomy. A specimen of the tumor was collected and processed for RQ-PCR analysis. The final histopathology reported a 2 cm × 1 cm G2 endometrioid EC with a 30% myometrial deep invasion (FIGO Stage 1A) (Figure 2A). Concerning the clinicopathological parameters routinely assessed in EC, lymphovascular space invasion (LVSI) was negative, both estrogen receptor (ER) and progesterone receptor (PR) were positive, p53 was positive, whereas PTEN was negative. The patient was considered as low risk for recurrence; therefore, no adjuvant treatment was recommended, and follow-up was started.

In July 2015, the patient experienced a dull pelvic pain, starting from the right lower quadrant, migrating to the right flank. Physical examination revealed tenderness in the right lower quadrant. An abdominal ultrasound scan (US) revealed a right-sided grade 2 hydronephrosis. A total body computer tomography (CT) and a pelvic MRI showed two solid lesions: a 5 cm × 3 cm × 3.8 cm recurrence at the vaginal vault with infiltration of the left wall of the bladder, near the ureterovesical junction, and another lesion (16 mm × 8 mm) surrounding the right pelvic ureter that caused hydronephrosis. The patient underwent cystoscopy with multiple biopsies of the left bladder wall, which were positive for recurrence of EC. The patient started six cycles of a three-weekly schedule of carboplatin and paclitaxel.

In January 2016, after the first cycle of chemotherapy, the patient developed an enterovaginal fistula and subsequently an ileal subocclusion. The patient underwent ileal resection, omentectomy, as there was macroscopic disease, and fistula repair. The final histopathology was positive for EC recurrence at ileum, omentum, and vaginal wall (Figure 2B). The patient continued chemotherapy. The local Ethical Committee of AOU Careggi (Florence, Italy) approved this study. Patient has given her written informed consent, in accordance with the Declaration of Helsinki.

## Background

Endometrial cancer (EC) is the most common malignancy of the female reproductive tract with an increasing incidence (1). In the last 10 years, EC has been generally divided into three risk groups: high-, intermediate-, and low-risk group (2). Several prognostic factors, namely FIGO Stage, depth of myometrial invasion, tumor differentiation grade, tumor histotype, and lymphovascular space involvement, are currently assessed in EC to classify the patients into the three risk groups. Moreover, large clinical trials, such as PORTEC-1 and GOG-99, have established other risk factors, i.e., older age and tumor size (3, 4). Several molecular markers have been reported as predictors of outcome in EC and are likely to contribute to future personalized therapy. However, these molecules are still under investigation and currently not in clinical practice (2).

Despite a rather favorable prognosis in low-risk patients, 4–10% of tumors will recur. The vagina is the most commonly affected site by local recurrence (5). Vaginal brachytherapy is a recommended adjuvant therapy in only intermediate- and in high-risk patient with negative nodes (2). Indeed, prophylactic vaginal irradiation in the low-risk group is not recommended, though still under debate (6, 7). A multicentre randomized study, on vaginal brachytherapy in FIGO Stage I low-risk EC, reported a lower rate of vaginal recurrence in the treatment group compared with the controls, although the difference was not statistically significant (7). On the contrary, in ASTEC/EN5 trial, no benefit of vaginal brachytherapy in low-risk patients was found (8). In this group of patients, the average risk of recurrence after surgery alone is low and adjuvant retrotranscribed (RT) does not increase survival outcomes. Nevertheless, within the low-risk patients, there might be subgroups of patients who may benefit from vaginal brachytherapy and/or targeted therapies. Therefore, additional prognostic tools are needed to improve the definition of the risk of recurrence in patients with EC.

## Discussion

Our patient was 51-year-old women when cancer was diagnosed and most of the prognostic factors were known prior to surgery by endometrial biopsy and pelvic RMI. Therefore, the patient was treated as a low-risk EC with surgery alone and a close follow-up. Clinicopathological parameters assessed after surgery confirmed the low-risk status of the patient. She had a G2 endometrioid EC, FIGO Stage IA, both ER and PR were highly expressed, and LVSI was negative. Indeed, in patients with EC, higher level of ER and PR predict favorable survival (9), and the presence of LVSI is currently considered as an independent prognostic factor associated with an increased risk of pelvic lymph node metastases and/or relapse of disease (2). Finally, even though p53 hotspot mutations are significantly more frequently found in serous ECs than in endometrioid EC, our patient had p53 positive (10). In some observational studies, mutations of p53 have been associated with aggressive forms of EC (11, 12), although in the latest guidelines, the presence of p53 mutations is not considered in the classification of high-risk patients (2).

Six months after surgery, our patient developed an early vescico-vaginal recurrence and few months later a subsequent involvement of vaginal wall, ileum, and omentum, despite having already started chemotherapy.

Various molecular biomarkers have been investigated in order to improve the detection of women with increased risk of nodal metastases and/or recurrence within each risk group, and consequently to tailor surgical staging procedures and adjuvant therapies. Identifying the key factors/pathways responsible for the aggressiveness of EC is urgently needed in order to address the alarming trend of decreasing survival. The Cancer Genome Atlas Research Network has recently published an integrated genomic, transcriptomic, and proteomic characterization of 373 endometrial carcinomas (10). The Cancer Genome Atlas database is a large-scale genomic database that primarily envisions somatic changes in cancers, including EC. The final goal of the study was to tailor patient treatment in a personalized manner and drive customization of disease care by implementing tumor genetic information. The analysis provided insights into disease biology and diagnostic classification of EC into four categories: POLE ultramutated, microsatellite instability hypermutated, copy-number low, and copy-number high. Nevertheless, these molecular factors have not been incorporated into any classifications for treatment and follow-up of EC since they are still under investigation.

Finally, our group contributed to this challenge defining the role of LH/hCG-receptor (LH/hCG-R)/luteinizing hormone (LH) in EC tumor progression. Our researches have demonstrated that (i) LH/hCG induces an *in vitro* invasive phenotype, through the activation of LH/hCG-R and hence of protein kinase A (PKA) (13); (ii) LH/hCG-R mRNA is expressed in a small cohort primary ECs (14); (iii) primary treatment with Gn-RH analogs (aimed to decrease the levels of serum LH) for 6 years in a patient affected by EC with contraindications to surgery, was associated with no evidence of progression of the disease throughout the study period (15); (iv) LH/hCG-R in a preclinical mouse model of EC behaves as a prometastatic molecular device (16).

**Figure 3:**
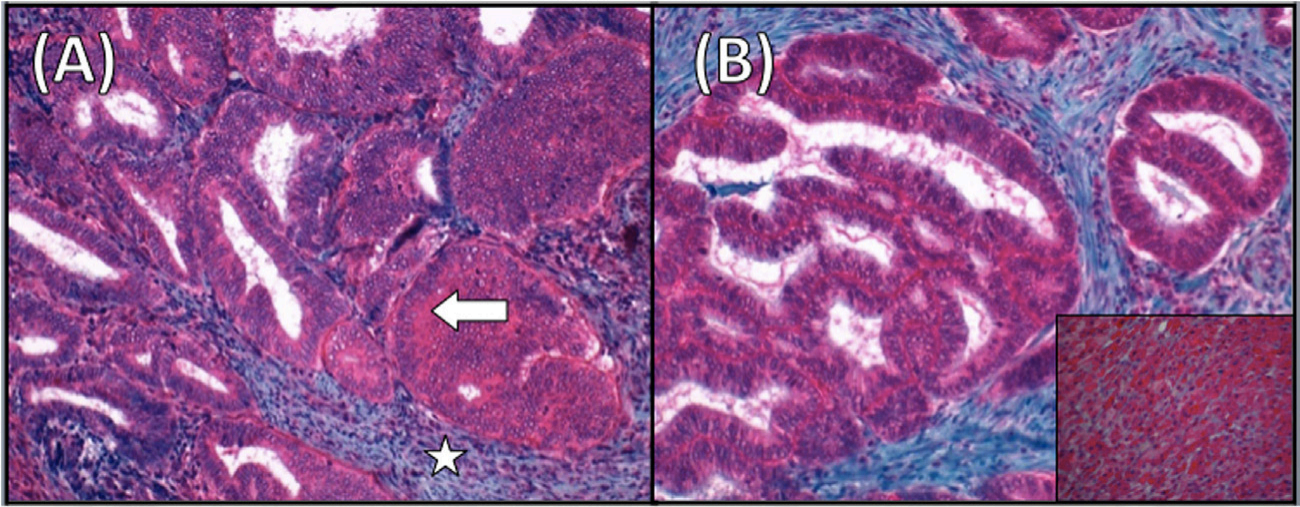
**Luteinizing hormone receptor (LH/hCG-R) staining in endometrial cancer (EC)**. **(A)** Immunohistochemistry (IHC) for LH/hCG-R in the EC specimen. Arrow indicates LH/hCG-R staining at the cytoplasmic level. The surrounding endometrial stroma (star) is negative for staining (magnification ×20). **(B)** Immunostaining for LH/hCG-R showing strong positivity also in the ileal metastases. Inset showing a section of corpus luteum used as positive control (magnification ×20).

In this paper, we describe an LH/hCG-R expression in cancer specimens collected at primary diagnosis and at relapse and immunohistochemical (IHC) analysis for LH/hCG-R in all paraffinated cancer specimens collected at primary diagnosis and relapse (Figure 3). The expression turned out to be high in both the analyses and in all specimens.

### LH/hCG-R Analysis: Methods

(1) RNA isolation, reverse transcription, and RQ-PCR analysis. Neoplastic tissues were conserved in RNA-later (Invitrogen) and then homogenized in Trizol Reagent (Invitrogen). The RNA integrity was assessed with the Agilent 2100 Bioanalyzer. One microgram of RNA was RT using random primers and reverse transcriptase (SuperScript II Reverse Transcriptase, Invitrogen) according to manufacturer’s protocol. cDNA was used as template for RQ-PCR analysis with SYBR green fluorescent dye (Power SYBR^®^ Green, PCR master mix, and Applied Biosystems). The relative expression of genes was calculated with the comparative threshold cycle method. The GAPDH gene was used as a standard reference, as already reported (17). A pool of normal mucosa was used as calibrator for LH/hCG-R expression. Primers used were the following: LH/hCG-R sense TGCCTACCTCCCTGTCAAAG, antisense TTGAGGAGGTTGTCAAAGG; *a-sma* sense GACGAAGCACAGAGCAAAAGAG, antisense TGGTGATGATGCCATGTTCTATCG-3′; *vimentin* sense CCTTGAACGCAAAGTGGAAT*;* antisense TTTGGACATGCTGTTCCTGA (18).
(2) IHC analysis. IHC staining was performed on 3 μm thick serial sections cut from formalin-fixed and paraffin-embedded tissues. As primary antibody we used rabbit polyclonal against luteinizing hormone receptor (LHR) (Novus Biologicals, Littleton, CO, USA) at dilution 1:50 overnight at 4°C. Antigen retrieval is performed in thermostat bath (PT Link, Pre-Treatment Module Dako) at 97°C with Citrate buffer 10 mM pH 6 for 8 min. For chromogenic detection, ultraView Universal RED detection kit (Ventana Medical Systems, Tucson, AZ, USA) was used. The sections were lightly counterstained with Mayer’s hematoxylin. A negative control sample was performed by omitting the primary antibody. Sections of corpus luteum were used as positive control. The control sections were treated in parallel with the samples in the same run.

### LH/hCG-R Analysis: Results

RQ-PCR analysis of EC specimen showed a strong hyper expression of mRNA for LH/hCG-R, just above 180-fold than normal mucosa (mean Ct values are reported in Table 1).

**Table 1:** **Evaluation by RQ-PCR of the transcript levels of LHR transcript and of fibroblast markers in a primary endometrial cancer specimen**.

	Ct values GAPDH (mean + SD)	Ct values LHR (mean + SD)	Ct values a-SMA (mean + SD)	Ct values vimentin (mean + SD)
Reference	14.770 ± 0.055	36.163 ± 0.723	25.771 ± 0.110	28.083 ± 0.143
Patient	22.126 ± 0.100	36.015 ± 0.625	32.220 ± 0.152	28.083 ± 0.143

To further characterize the primary tissue sample, we performed a RQ-PCR to determine the expression of fibroblast markers (vimentin and α-SMA). The results showed low expression of vimentin and α-SMA, confirming that the tissue sample contain almost exclusively tumor cells (mean Ct values are reported in Table 1).

Gene expression levels were evaluated by RQ-PCR using the primer pairs reported in materials and methods. Table shows the mean ± SD of Ct values of the different transcripts, as well as of the housekeeping gene GAPDH.

## Concluding Remarks

Patients with EC are defined as low/intermediate/high risk of recurrence based on clinicopathological prognostic factors (2). However, such management may lead to undertreatment and, among the low-risk group; the most frequent consequence is local vaginal relapse (7).

Previous studies by our group *in vitro* and in preclinical model have reported that the over-expression of the LH/hCG-R increases the ability of EC cells to undergo local invasion and metastatic spread (13, 14, 16). In this paper, we described high levels of expression of LH/hCG-R at both mRNA and protein levels in a patient with EC who experienced early relapses, even though she was defined as low risk according to the current guidelines. For this reason, in our opinion, it may be possible to claim that there is a relationship between high LH/hCG-R expression and biological aggressiveness of EC.

In conclusion, our report suggests that LH/hCG-R expression in EC specimens, together with other clinicopathological markers, should be taken into account in order to assess the risk profile in patients with EC. Further clinical trials are warranted in order to validate LH/hCG-R as biomarker in low-risk EC.

## Author Contributions

IN has contributed to the concept of this manuscript, analysis of literature, and revision of the paper. FS has made contributions to execution of surgery, the concept of this manuscript, analysis of literature, and revision of the paper. LM has contributed to the concept of this manuscript, analysis of literature, and revision of the paper. EP has contributed to analysis of histology, the concept of this manuscript, and revision of the paper. SP has contributed to the concept of this manuscript, analysis of RQ-PCR, and revision of the paper. VG has contributed to the concept of this manuscript, analysis of literature, and revision of the paper. TL has contributed to the concept and revision of this manuscript. DM has contributed to analysis of histology, the concept of this manuscript, and revision of the paper. GB has contributed to analysis of histology, the concept of this manuscript, and revision of the paper. FM has contributed to the concept and revision of this manuscript. AA has contributed to the concept of this manuscript, analysis of literature, and revision of the paper. MF has made contributions to execution of surgery, the concept of this manuscript, analysis of literature, and revision of the paper.

## Conflict of Interest Statement

The authors declare that the research was conducted in the absence of any commercial or financial relationships that could be construed as a potential conflict of interest. The reviewer ST and handling editor declared their shared affiliation, and the handling editor states that the process nevertheless met the standards of a fair and objective review.
